# Self-reported suboptimal sleep and receipt of sleep assessment and treatment among persons with and without a mental health condition in Australia: a cross sectional study

**DOI:** 10.1186/s12889-021-10504-6

**Published:** 2021-03-06

**Authors:** Alexandra P. Metse, Caitlin Fehily, Tara Clinton-McHarg, Olivia Wynne, Sharon Lawn, John Wiggers, Jenny A. Bowman

**Affiliations:** 1 University of Newcastle, University Drive, Callaghan, NSW 2308 Australia; 2 University of the Sunshine Coast, 90 South Street, Murdoch, WA 6150 Australia; 3 Hunter Medical Research Institute, Lot 1 Kookaburra Circuit, New Lambton Heights, NSW 2305 Australia; 4 Murdoch University, 90 Sippy Downs Drive, Sippy Downs, QLD 4556 Australia; 5 Flinders University, Sturt Rd, Bedford Park, SA 5042 Australia; 6 Hunter New England Population Health, Longworth Avenue, Wallsend, NSW 2287 Australia

**Keywords:** Mental health conditions, Sleep, Assessment, Treatment, Care provision, Health risk behaviour

## Abstract

**Background:**

Poor sleep and poor mental health go hand in hand and, together, can have an adverse impact on physical health. Given the already disproportionate physical health inequities experienced by people with a mental health condition worldwide, the need to consider and optimise sleep has been highlighted as a means of improving both physical and mental health status.

Sleep recommendations recently developed by the United States’ National Sleep Foundation incorporate a range of sleep parameters and enable the identification of ‘suboptimal’ sleep. Among community-dwelling persons with and without a 12-month mental health condition in Australia, this study reports: [1] the prevalence of ‘suboptimal’ sleep and [2] rates of sleep assessment by a health care clinician/service and receipt of and desire for sleep treatment.

**Methods:**

A descriptive study (*N* = 1265) was undertaken using self-report data derived from a cross-sectional telephone survey of Australian adults, undertaken in 2017.

**Results:**

Fifteen per cent (*n* = 184) of participants identified as having a mental health condition in the past 12 months. Across most (7 of 8) sleep parameters, the prevalence of suboptimal sleep was higher among people with a mental health condition, compared to those without (all *p* < 0.05). The highest prevalence of suboptimal sleep for both groups was seen on measures of sleep duration (36–39% and 17–20% for people with and without a mental health condition, respectively). In terms of sleep assessment and treatment, people with a mental health condition were significantly more likely to: desire treatment (37% versus 16%), have been assessed (38% versus 12%) and have received treatment (30% versus 7%).

**Conclusions:**

The prevalence of suboptimal sleep among persons with a mental health condition in Australia is significantly higher than those without such a condition, and rates of assessment and treatment are low for both groups, but higher for people with a mental health condition. Population health interventions, including those delivered as part of routine health care, addressing suboptimal sleep are needed.

**Supplementary Information:**

The online version contains supplementary material available at 10.1186/s12889-021-10504-6.

## Background

Sleep of poor quality and/or inadequate/excessive duration is prevalent among adults in Australia (33–45% [1]) and other high income countries (Canada: 35% [2]), and is associated with significant preventable illness and death [3–6]. Poor sleep increases the risk of certain cardiovascular and cerebrovascular diseases and cancer [5, 7–9] and also onset and relapse from mental health conditions including depression, anxiety and psychosis [10, 11]. Accordingly, poor sleep has been recognised as a health risk behaviour and a priority for public health research and intervention [12].

People with a mental health condition in Australia have a reduced life expectancy compared to the general population: 16 years less for men and 12 years less for women [13], with 78% of this excess mortality attributed to preventable chronic conditions including cardiovascular disease, cardiometabolic disease and cancer [14, 15]. A recent Lancet Psychiatry Commission identified poor sleep as a significant contributor to the physical health inequity experienced by this group, in addition to the high prevalence of other health risk behaviours including poor nutrition and physical inactivity [15]. The high level of comorbidity between poor sleep and mental health conditions (between 50 and 80% depending on diagnosis and setting [11, 15]) is reported to result in both greater physical and mental health burden, exacerbating symptoms and reducing treatment efficacy for conditions such as low mood, anxiety and psychosis [11, 13, 16, 17]. It has been acknowledged that poor sleep may reduce a person’s capacity to lead ‘a meaningful, contributing life’, negatively impacting opportunities for employment and for social participation and connection with others more generally [11]. Poor sleep can be effectively treated among people with mental health conditions [16] with such treatment leading to improvements in physical and mental wellbeing [16, 18–20].

Sleep duration and quality recommendations published by the United States’ (US) National Sleep Foundation (NSF) in 2015 [21] and 2017 [22] respectively, have been recognised by the Australasian Sleep Association [23]. Different sleep duration and quality criteria are identified for young (18–25 years), middle (26–64 years) and older (65+ years) adults, accounting for normal changes to sleep that occur across adulthood (see Supplementary Table 1) [21, 22]. The NSF criteria represent a consensus on indicators of sleep considered to be ‘suboptimal’ for the general population across parameters including duration, onset latency, awakenings after onset, efficiency and naps (see Supplementary Table 1) [21, 22].

Two population-level studies have assessed the prevalence of self-reported ‘suboptimal’ sleep, defined according to the NSF criteria [2, 24] for the general population. The most recent, a cross-sectional study of Australian adults (*N* = 1265) undertaken in 2017, examined prevalence across 8 sleep parameters [24] and found 42% of participants met NSF criteria for suboptimal sleep: 19% on just one parameter and 24% on 2 or more parameters. The prevalence of suboptimal sleep was highest on measures of sleep duration (20–23%) [24]. The second study, a series of cross-sectional telephone surveys of Canadian adults (*N* = 10, 967) undertaken between 2007 and 2013, assessed only sleep duration and found that 35% reported a duration either shorter or longer than that recommended [2]. To date, however, no population-level study has examined the prevalence of suboptimal sleep defined according to NSF sleep duration and quality criteria [21, 22] among people with a mental health condition, and none have compared the prevalence of suboptimal sleep between people with and without such a condition.

Routine assessment of health risk behaviours (such as poor nutrition and physical inactivity) by health care providers increases the likelihood of detection and provision of evidence-based intervention [25, 26]. Guidelines, such as those in place for general practice in Australia [27], recommend a standardised and systematic approach to the provision of assessment and related preventative health treatment for common health risk behaviours. Systematic review evidence, however, indicates disparities in the provision of such preventative health treatment between people with and without a mental health condition [28, 29]. In Australia [30] and elsewhere [9] the need for routine assessment and provision of treatment for poor sleep is recognised. Survey and medical record audit data, however, for general population samples in Australia (< 10%) [24] and elsewhere (< 25%) [31] suggest such assessment and treatment rarely occurs.

There has been very little investigation of the provision of assessment and treatment for suboptimal sleep among people with a mental health condition, with the only two studies undertaken in the United Kingdom (UK) [11, 32]. The most recent, a cross-sectional survey of mental health outpatients 18 to 30 years of age with early psychosis (*n* = 60) [11], measured the prevalence of comorbid sleep disorders (using a validated structured diagnostic interview, sleep diaries, and actigraphy) and the receipt of sleep treatments such as Cognitive Behaviour Therapy for Sleep (CBT-S) and medication. Eighty per cent of participants had a least one sleep disorder: over half of the disorders (n = 60, 53%) had been discussed with a clinician and nearly a third had received treatment (*n* = 34, 30%). Treatment according to clinical guidelines was rare, occurring for only 8% of disorders (*n* = 13). The second study, a cross-sectional survey undertaken in 2015 of staff (*n* = 19) and consumers (*n* = 73) of a secondary mental health care setting, found 64% of consumers experienced poor sleep (assessed using the Pittsburgh Sleep Quality Index), of which 61% had not been offered any sleep treatment [32]. The most common treatment received by such consumers was medication prescription (32%), while CBT-S was received by only 6% of consumers. Two recent qualitative studies involving persons with schizophrenia spectrum disorders (*N* = 15 [33]; *N* = 10 [34]) recruited from community and health care settings in the UK, found treatment for sleep difficulties was viewed very positively and desired among participants. No research to date has compared the provision of sleep assessment and treatment for people with and without a mental health condition, nor their desire for sleep care.

Given the existing gaps in the literature, a study was undertaken to:

1. Examine the prevalence of self-reported suboptimal sleep, categorised according to the NSF sleep duration and quality criteria [21, 22], for adult with a mental health condition as compared to those without; and
2. Identify the rates of sleep assessment by health care clinicians/services and receipt of and desire for sleep treatment, for adults with a mental health condition as compared to those without.

## Methods

The methods for this study have been reported elsewhere [24] and are summarised below.

### Design and setting

A descriptive study was undertaken using data from the National Social Survey (NSS): an Australia-wide cross-sectional telephone survey.

### Data collection

The NSS was administered by trained interviewers at Central Queensland University. The interviews were undertaken every day between 17th July and 23rd August, 2017. Interviews lasted, on average, 38 min [24].

### Sample and recruitment

Landline and mobile numbers were called. Random digit dialling and number selection approaches were adopted. Eligible participants were: 18+ years old, an Australian resident, and contactable by telephone. Informed consent was obtained verbally prior to progressing with the interview.

### Measures

The subset of survey items assessed as part of the current study included: participant demographic information, including mental health status; sleep parameters; and assessment of sleep and receipt of and desire for sleep treatment.

#### Participant demographic information and mental health status

Demographic information collected included: gender, age, geographic distribution, Aboriginal and/or Torres Strait Islander origin, employment status, educational attainment, state or territory of residence and marital status [24].

To assess mental health status, participants were asked: ‘Have you received a diagnosis or treatment for any of the following mental health conditions in the past 12 months: depression, anxiety, schizophrenia, other form of psychosis, bipolar disorder, personality disorder, substance use disorder, other mental health condition, none?’ [35]. Multiple responses were permitted.

#### Sleep parameters

Items were created to align with the NSF sleep duration and quality recommendations [21, 22] and have been utilised and described previously [24]. Sleep duration (hours) on weekdays/workdays and weekends/nonworkdays was assessed. Measures of sleep quality included: sleep onset latency (minutes), number and duration (minutes) of awakenings after sleep onset, sleep efficiency (%; see [24] for approach to calculation), and number (per day) and duration (minutes) of naps.

#### Assessment of sleep and receipt of and desire for sleep treatment

Separate items examined, in the past 12-months, rates of sleep assessment by a health care clinician/service and receipt of sleep treatment [24]. Where treatment had been received, a further item assessed the type [24]. Another item assessed desire for sleep support/treatment, with response options on a Likert scale [24].

### Variable transformation

In accordance with NSF criteria, sleep measures were reduced to two levels (suboptimal, may be appropriate/appropriate; see [24] for full details).

For the purpose of association analyses, the following variables were also reduced to two levels: mental health status (yes, no), sleep assessment (yes, no), sleep treatment (yes, no), and desire for sleep treatment (no [‘neither agree nor disagree’/ ‘disagree’/ ‘strongly disagree’], yes [‘strongly agree’/‘agree’]) [24].

### Analyses

Descriptive statistics were used to summarise, according to mental health status, the prevalence of suboptimal sleep, assessment rates and receipt of and desire for treatment. Rates of assessment and receipt of treatment were also summarised for those meeting criteria for suboptimal sleep on at least one parameter, according to mental health status. Chi-square analyses were used to assess univariate associations between mental health status and (1) parameters of suboptimal sleep, (2) rates of sleep assessment, and (3) receipt of and (4) desire for sleep treatment.

## Results

### Sample

Twelve hundred and sixty-five people, out of the 5450 contacted, completed the survey (23% response rate). Reasons for non-completion included: refusal (2856; 68%), being non-contactable (804; 19%), commenced but did not finish (18, 0.004%) and ‘other’ (507, 12%). The response rate of 23% is comparable to that achieved previously for the NSS [36] and similar to other national telephone surveys [37].

Table 1 summarises participant demographic and health status data. Fifteen per cent (*n* = 184) of respondents identified to have been diagnosed with or treated for a mental health condition in the past 12 months, with depression (*n* = 128) and anxiety (*n* = 108) the most common types of conditions. People with a mental health condition, compared to those without, were more likely to be female (60% versus 51%; *X*^2^ [1] = 5.0, *p* < 0.05), less than 65 years old (73% versus 64%; *X*^2^ [2] = 6.3, *p* < 0.05), and not currently have full or part time employment (60% versus 43%; *X*^2^ [1] = 18.2, *p* < 0.001). There were no significant differences between people with and without a mental health condition in terms of education level, state or territory of residence, geographic distribution, or the proportion who identified to be of Aboriginal and/or Torres Strait Islander origin (Table 1).

**Table 1:** Demographic Information for Participants With and Without a Mental Health Condition

	Persons with a mental health condition (*n* = 184)	Persons without a mental health condition (*n* = 1081)	Total (*N* = 1265)
**Marital status**^a^			
Single (never married)	44 (24)	189 (18)	233 (18)
Widowed	18 (10)	88 (8)	106 (8)
Divorced	15 (8)	53 (5)	68 (5)
Separated not divorced	13 (7)	21 (2)	34 (3)
Married	74 (40)	639 (59)	713 (56)
De facto	18 (10)	86 (8)	104 (8)
No response	2 (1)	5 (1)	7 (1)
**Gender**^a^			
Male	73 (40)	525 (49)	598 (47)
**Age**^a^			
18–25	20 (11)	94 (9)	114 (9)
26–64	114 (62)	586 (55)	700 (56)
65+	50 (27)	393 (37)	443 (35)
No response	0 (0)	8 (1)	8 (1)
**Identify to be Aboriginal and/or Torres Strait Islander**^a^			
Yes	5 (3)	22 (2)	27 (2)
No	178 (97)	1054 (98)	1232 (97)
Unsure/ no response	1 (1)	5 (1)	6 (1)
**Highest level of education (complete or incomplete)**^a^			
Pre-school	0 (0)	3 (0)	3 (0)
Infants/primary school	3 (2)	17 (2)	20 (2)
Secondary/high school	62 (34)	332 (31)	394 (31)
Technical or further educational institution (e.g. TAFE colleges)	32 (17)	211 (20)	243 (19)
University or other higher educational institution	86 (47)	512 (47)	598 (47)
No schooling/no response	1 (1)	6 (1)	7 (1)
**Employment status** ^a,&^			
Employed full-time	42 (23)	393 (36)	435 (34)
Employed part-time/casual	32 (17)	223 (21)	255 (20)
Unemployed	20 (11)	35 (3)	55 (4)
Retired/pension	73 (40)	383 (35)	456 (36)
Student	6 (3)	23 (2)	29 (2)
Home duties	8 (4)	17 (2)	25 (2)
No response	3 (2)	7 (0)	10 (1)
**State or territory residing**^a^			
Australian Capital Territory (ACT)	0 (0)	19 (2)	19 (2)
New South Wales (NSW)	51 (28)	323 (30)	374 (30)
Northern Territory (NT)	1 (1)	11 (1)	12 (1)
Queensland (QLD)	45 (25)	236 (22)	282 (22)
South Australia (SA)	20 (11)	86 (8)	106 (8)
Tasmania (TAS)	4 (2)	30 (3)	34 (3)
Victoria (VIC)	39 (21)	268 (25)	307 (24)
Western Australia (WA)	23 (13)	104 (10)	127 (10)
No response	0 (0)	4 (0)	4 (0)
**Geographic distribution**^a^			
City	100 (54)	562 (52)	662 (52)
Town	34 (19)	224 (23)	278 (22)
Rural area	49 (27)	271 (25)	320 (25)
Unsure/ no response	1 (1)	4 (0)	5 (0)
**Type of mental health condition (12-month)**^a,^*			
Depression	128 (70)	–	128 (10)
Anxiety disorder	108 (59)	–	108 (9)
Schizophrenia	6 (3)	–	6 (1)
Bi-polar disorder	9 (5)	–	9 (1)
Personality disorder	7 (4)	–	7 (1)
Other form of psychosis	3 (2)	–	3 (0)
Substance use disorder	2 (1)	–	2 (0)
Other mental health condition	20 (11)	–	18 (1)
None of the above	n/a	–	1058 (84)
No response	0 (0)	–	23 (2)

^a^ Number (%); ^b^ Mean (standard deviation); ^&^ Primary employment status; * Multiple responses permitted

### Prevalence of suboptimal sleep

The highest prevalence of suboptimal sleep was found on measures of sleep duration and onset latency for both people with and without a mental health condition (Table 2). Across all except one parameter (number of naps per day), the prevalence of suboptimal sleep was at least twice as high among people with a mental health condition, compared to those without (Table 2).

**Table 2:** Prevalence of Suboptimal Sleep, Rates of Assessment and Receipt of and Desire for Sleep Treatment

		Persons with a mental health condition	Persons without a mental health condition	Total	
		Number (%)	Number (%)	Number (%)	
**Sleep duration** *(on weekdays/workdays)*^1^					*X*^2^ [1] = 31.1***
Appropriate		62 (34)	418 (39)	480 (39)	
May be appropriate		50 (27)	430 (41)	480 (39)	
Suboptimal		71 (39)	213 (20)	284 (23)	
* Inadequate*		*55 (78)*	*175 (82)*	*230 (81)*	
* Excessive*		*16 (23)*	*38 (18)*	*54 (19)*	
**Sleep duration** *(on weekends/non-work days)*^1^					*X*^2^ [1] = 37.0***
Appropriate		60 (33)	515 (49)	575 (46)	
May be appropriate		56 (31)	368 (35)	424 (34)	
Suboptimal		66 (36)	179 (17)	245 (19)	
*Inadequate*		*48 (73)*	*127 (71)*	*175 (71)*	
*Excessive*		*18 (27)*	*52 (29)*	*70 (29)*	
**Sleep onset latency**^2^					*X*^2^ [1] = 32.4***
Appropriate		118 (66)	874 (82)	992 (80)	
May be appropriate		11 (6)	67 (6)	78 (6)	
Suboptimal		50 (28)	127 (12)	177 (14)	
**Awakenings (> 5 min) per night**^3^					*X*^2^ [1] = 19.0***
Appropriate		90 (50)	719 (67)	809 (65)	
May be appropriate		59 (32)	268 (25)	327 (26)	
Suboptimal		33 (18)	85 (8)	118 (9)	
**Wake after sleep onset**^1^					*X*^2^ [1] = 13.1***
Appropriate		130 (72)	894 (84)	1024 (82)	
May be appropriate		29 (16)	108 (10)	134 (11)	
Suboptimal		22 (12)	64 (6)	86 (7)	
**Sleep efficiency**^&,4^					*X*^2^ [1] = 9.0**
Appropriate		119 (71)	845 (84)	964 (76)	
May be appropriate		27 (16)	114 (11)	141 (11)	
Suboptimal		21 (13)	53 (5)	74 (6)	
**Nap frequency (days/week)**^5^					
Appropriate		13 (7)	55 (5)	68 (5)	
May be appropriate		171 (93)	1021 (95)	1192 (95)	
Suboptimal		N/A	N/A	N/A	
**Number of naps per day**^6^					*X*^2^ [1] = 0.1
Appropriate		13 (7)	55 (5)	68 (5)	
May be appropriate		170 (92)	1011 (94)	1181 (94)	
Suboptimal		1 (1)	8 (1)	9 (1)	
**Nap duration**^7^					*X*^2^ [1] = 17.2***
Appropriate		N/A	N/A	N/A	
May be appropriate		166 (90)	1035 (97)	1201 (96)	
Suboptimal		18 (10)	34 (3)	52 (4)	
**In the past 12-months, have you been asked about the duration and/or quality of your sleep from a health care clinician or service?**					*X*^2^ [1] = 77.3***
Yes		70 (38)	133 (12)	203 (16)	
No		30 (16)	157 (15)	187 (15)	
Nil contact with a health care clinician or service in the past 12-months		84 (46)	789 (73)	873 (69)	
Unsure/no response		0 (0)	2 (0)	2 (0)	
**In the past 12-months, have you received any intervention or recommendations to improve the duration and/or quality of your sleep from a health care clinician or service?**					*X*^*2*^ [1] = 93.5***
No		44 (24)	217 (20)	261 (21)	
Yes		56 (30)	75 (7)	131 (10)	
*Sleep medication recommended or prescribed*^*#*^		*32 (57)*	*21 (28)*	*56 (43)*	
*Sleep aids such as CPAP machine or nasal strips recommended or prescribed*^*#*^		*10 (18)*	*22 (29)*	*32 (24)*	
*Sleep hygiene education*^*#*^		*10 (18)*	*12(16)*	*22 (17)*	
*Psychosocial support such as Cognitive Behavioural Therapy (CBT)*^*#*^		*5 (9)*	*4(5)*	*9 (7)*	
*Other support (referral to specialist service, medication advice,* etc.*)*^*#*^		*16 (29)*	*23 (31)*	*39 (30)*	
Nil contact with a health care clinician or service in the past 12-months		84 (46)	789 (73)	873 (69)	
**“I would like to receive support to improve the duration and/or quality of my sleep from a health care clinician or service.”**					*X*^2^ [1] = 42.6***
Strongly agree		27 (15)	41 (4)	68 (6)	
Agree		40 (22)	132 (12)	172 (14)	
Neither agree nor disagree		29 (16)	136 (13)	165 (13)	
Disagree		63 (34)	525 (49)	588 (47)	
Strongly disagree		22 (12)	232 (22)	254 (20)	
Unsure/no response		3 (2)	15 (1)	18 (1)	

^1^Missing: n = 21; ^2^Missing: n = 18; ^3^Missing: *n* = 11;^4^Missing: *n* = 86; ^5^Missing: n = 5;^6^Missing: n = 7; ^7^Missing: n = 12; ^&^Sleep efficiency on weekdays; ^#^Multiple responses permitted; N/A: not applicable

* *p* < 0.05; ** *p* < 0.01; *** *p* < 0.001

People with a mental health condition were also more likely to meet suboptimal criteria on a greater number of parameters. Sixty-five per cent (*n* = 120) of people with a mental health condition met criteria for suboptimal sleep on at least one parameter and 23% (*n* = 43) on three or more, compared to 38% (*n* = 414) and 8% (*n* = 88) of those without, respectively.

### Assessment of sleep, and receipt of and desire for sleep treatment

People with a mental health condition were more likely than those without to report that their sleep had been assessed (38 and 12%, respectively; *X*^2^ [1] = 77.3, *p* < 0.001) and also to have received sleep treatment (30 and 7%, respectively; *X*^2^ [1] = 93.5, *p* < 0.001). Of those who had received treatment, 57% (*n* = 32) of people with a mental health condition were recommended or prescribed medication compared to 28% (*n* = 21) of those without. Similar proportions of people with and without a mental health condition (18% versus 16%) were provided with sleep hygiene information, and less than 10% of both groups received psychosocial support such as Cognitive Behaviour Therapy for sleep (Table 2). CPAP or nasal strips was recommended to or used by a higher proportion of people without a mental health condition (29% versus 18%).

The proportion of participants who desired sleep treatment was twice as high among those with a mental health condition compared to those without (36% versus 16%; *X*^2^ [1] = 42.6, *p* < 0.001; Table 2).

**Fig. 1:**
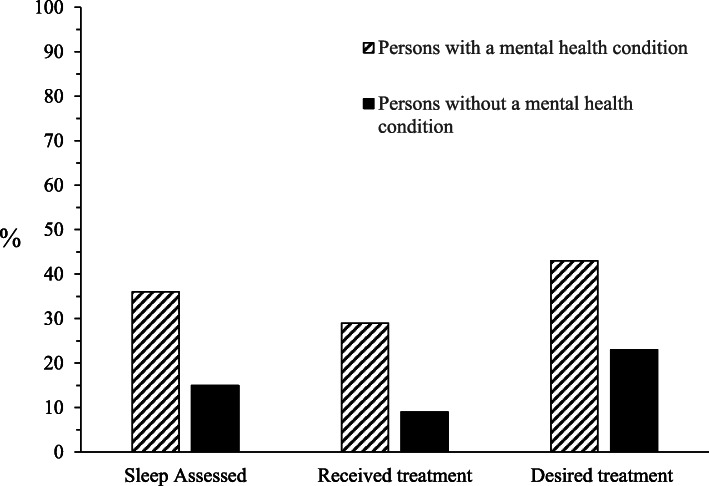
Proportion of Participants with Suboptimal Sleep who Received Assessment and Treatment, and Desired Sleep Treatment

Among participants with suboptimal sleep on at least one parameter, a higher proportion of those with a mental health condition compared to those without received both assessment (36% [*n* = 43] versus 15% [*n* = 62], respectively) and treatment (29% [*n* = 35] versus 9% [*n* = 37], respectively), and reported a desire for treatment (43% [*n* = 52] versus 23% [*n* = 95], respectively) (Fig. 1).

## Discussion

This is the first study to assess and compare the prevalence of suboptimal sleep, categorised according to the NSF sleep duration and quality criteria, and receipt of sleep assessment and treatment among people with and without a mental health condition in Australia. Across all but one parameter, the prevalence of suboptimal sleep among people with a mental health condition was at least twice that of those without such a condition. People with a mental health condition were also more likely to meet suboptimal criteria on a greater number of parameters. In addition, they were more likely to be assessed and receive treatment. Still, approximately two thirds of people with a mental health condition who were identified to meet suboptimal criteria on at least one sleep parameter had not had their sleep assessed, nor received any form of treatment; yet nearly half reported a desire for sleep treatment.

The finding that the prevalence of suboptimal sleep is higher among persons with a mental health condition than those without is comparable to previous research assessing rates of sleep problems and disorders between the two groups, without reference to the NSF duration and quality criteria [38, 39]. For example, a recent population-level survey of adults in low- and middle-income countries (*N* = 237,023) found reports of having difficulty sleeping (including falling asleep and waking through the night or too early) was significantly higher among those meeting criteria for a depressive episode (27%), compared to those not meeting such criteria (5%) [38]. The magnitude of difference in prevalence is also comparable (22% versus 19%) to that found in the current study.

The highest prevalence of suboptimal sleep for both groups was seen on measures of sleep duration, a finding consistent with previous research assessing the prevalence of suboptimal sleep duration among general population samples [2, 24] and people accessing inpatient psychiatry services [40]. Regarding inadequate and excessive sleep duration, the prevalence of inadequate sleep was higher on weekdays/workdays compared to weekends/nonwork days. By contrast, the prevalence of excessive sleep duration was higher on weekends/nonwork days compared to weekdays/workdays. People without a mental health condition had a greater difference in the prevalence of inadequate and excessive duration between workdays and weekdays compared to those with a mental health condition. This may be partially explained by the higher proportion of people without a mental health condition being in paid employment. Nevertheless, for both groups, the higher prevalence of inadequate sleep duration on weekdays/workdays and excessive duration on weekends/non work days is similar to that found in previous Australian population-level surveys [41] and suggestive of the presence of ‘social jetlag’, where people attempt to recover sleep debt accrued through the week due to occupational commitments on the weekends [42].

For both groups, the second highest prevalence of suboptimal sleep was on the measure of sleep onset latency. This finding is consistent with those of previous population-level studies of general populations [43] and small clinical studies of people with a mental health condition [44, 45]. However, these previous studies did not quantify the average onset latency or categorise duration using the NSF criteria, instead utilised self-report items such as ‘do you having difficulty falling asleep?’ [41]. Overall, our results are similar to that of relevant previous research in suggesting duration and onset latency are the parameters of sleep where both groups were most affected, and that rates of suboptimal sleep are disproportionately high among people with a mental health condition.

Contrary to review evidence suggesting disparities in routine assessment for other health risk behaviours [28], a higher proportion of people with a mental health condition had their sleep status assessed compared to those without. This finding may be explained by poor sleep being a common and prominent feature of many mental health conditions [46], and therefore assessment may have taken place in the context of mental health management rather than as a routine preventative health check. Another explanation may relate to a greater overall frequency of health service utilisation by people with a mental health condition [47], thereby increasing opportunity for assessment. Further, it has been suggested that psychiatrists are increasingly aware of indicators of comorbid sleep disorders in their patients [48], perhaps increasing likelihood of assessment and treatment. Nevertheless, similar to previous research suggesting provision of care for sleep is suboptimal [11, 31, 32], two thirds of people with a mental health condition who were experiencing suboptimal sleep, and 85% of those without such a condition had not been assessed. Notable proportions of both groups expressed a desire for sleep treatment.

People with a mental health condition were also more likely to have received a form of treatment, compared to those without such a condition. CBT-S is the first line treatment for suboptimal sleep [49–51]. Our results indicate that less than a third of people with (27%) and without (21%) a mental health condition in receipt of a form of sleep treatment were delivered such an intervention. Further, similar to previous research [32, 52], medication prescription was a common form of treatment received by both groups. For those with a mental health condition, the likelihood of receiving medication was notably higher (43% versus 28%). Research from Australia [53] and elsewhere [32–34] suggests people with severe mental health conditions have a preference and desire for the first line treatment, with medication desired only in rare instances for acute sleep problems.

Our findings related to the assessment and treatment of sleep suggest there is an unmet need for care. They support suggestions by the American Heart Association and other experts [9, 54] concerning the need for the development of policies and clinical practice guidelines to promote routine assessment and provision of first-line treatments for suboptimal sleep for the general population, and suggest further that these may need to be tailored to meet the needs and desires of people with a mental health condition. Additionally, sleep should be incorporated into existing preventative health screening and care guidelines [55]. Barriers to the implementation of guidelines will need to be addressed. For example, one UK survey assessed staff (*n* = 19) barriers to sleep assessment and treatment in secondary mental health care settings and identified deficits in knowledge of indicators/symptoms of suboptimal sleep a primary barrier [32].

The findings of the current study need to be considered in the context of a few limitations, some of which have been reported previously [24]. Firstly, our findings are based on self-report data. With regard to sleep measures, research has suggested a history of poor sleep may lead individuals to overestimate the extent of current difficulties [56]. Nonetheless, other research indicates self-report data for sleep are similarly predictive, compared to more objective measures, of sleep-related illness and death [57–59]. To strengthen conclusions that can be drawn, future studies should also collect objective sleep data. The measure used to determine mental health status was also self-report, considering diagnoses and treatment over a 12-month period. While the measure precluded assessment of current symptom severity, it has however, been used in previous population-level surveys assessing health risk behaviours [35, 60].

Secondly, the validity of the NSF criteria has not been assessed. They have, however, been recognised and their use recommended by peak bodies related to sleep and health ([23]; see [24] for further information).

Next, we did not assess for the presence of diagnosed sleep disorders (e.g. Obstructive Sleep Apnoea) or recent mental health treatment. Future research should explore the associations between suboptimal sleep (categorised according to NSF sleep duration and quality criteria) and the presence of various sleep disorders; in addition to relevant social, occupational and environmental factors [61, 62]. It should also assess and consider recent mental health treatment and its potential impact on sleep.

Lastly, in terms of sample representativeness, older adults (65+ years) and those living outside of urban areas were overrepresented, compared to Australian Bureau of Statistics data. Nevertheless, the impact on our findings was likely negligible, with non-significant differences found between outcomes using raw data and weighted data (accounting for overrepresentation).

## Conclusions

The prevalence of suboptimal sleep is approximately twice as high among people with a mental health condition in Australia, compared to those without such a condition. For people with and without a mental health condition, the highest rates of suboptimal sleep are seen on measures of duration and onset latency. Despite the potential for health services to play a role in both its prevention and treatment, findings suggest assessment and treatment to improve poor sleep may not be routinely provided to either group. Nearly twice the proportion of people with a mental health condition compared to those without desired treatment to improve their sleep, despite higher rates of assessment and treatment provision. Further, where treatment was provided, people with a mental health condition were more likely than those without such a condition to receive medication. Overall, considerable proportions of people experiencing suboptimal sleep in both groups were not assessed nor received treatment. Population health interventions to address suboptimal sleep are needed, including those delivered as part of routine health care.

## Supplementary Information

**Additional file 1: Supplementary Table 1.** NSF Criteria for Categorising Sleep Parameters as 'Appropriate', 'May be appropriate' and 'Suboptimal', According to Age.

## Data Availability

The materials and datasets generated and analysed during the current study are not publicly available. However, they are available from the corresponding author on reasonable request.

## Abbreviations

NSF: National Sleep Foundation
NSS: National Social Survey
US: United States
UK: United Kingdom
CBT-S: Cognitive Behaviour Therapy for Sleep
CPAP: Continuous Positive Airway Pressure

## References

[1] Adams R, Appleton S, Taylor A, McEvoy D, Antic N (2016). Report to the sleep Health Foundation 2016 sleep health survey of Australian adults.

[2] Chaput J-P, Wong S, Michaud I (2017). Duration and quality of sleep among Canadians aged 18 to 79. Health Rep.

[3] Lubetkin EI, Jia H (2018). Burden of disease due to sleep duration and sleep problems in the elderly. Sleep Health..

[4] Grandner MA, Hale L, Moore M, Patel NP (2010). Mortality associated with short sleep duration: the evidence, the possible mechanisms, and the future. Sleep Med Rev.

[5] Buxton OM, Marcelli E (2010). Short and long sleep are positively associated with obesity, diabetes, hypertension, and cardiovascular disease among adults in the United States. Soc Sci Med.

[6] Gallicchio L, Kalesan B (2009). Sleep duration and mortality: a systematic review and meta-analysis. J Sleep Res.

[7] Ding D, Rogers K, Macniven R, Kamalesh V, Kritharides L, Chalmers J (2014). Revisiting lifestyle risk index assessment in a large Australian sample: should sedentary behavior and sleep be included as additional risk factors?. Prev Med.

[8] Senaratna CV, English DR, Currier D, Perret JL, Lowe A, Lodge C (2016). Sleep apnoea in Australian men: disease burden, co-morbidities, and correlates from the Australian longitudinal study on male health. BMC Public Health.

[9] St-Onge M-P, Grandner MA, Brown D, Conroy MB, Jean-Louis G, Coons M, et al. Sleep duration and quality: impact on lifestyle behaviors and cardiometabolic health: a scientific statement from the American Heart Association. Circulation. 2016. 10.1161/CIR.0000000000000444 PMC5567876 27647451

[10] Hertenstein E, Feige B, Gmeiner T, Kienzler C, Spiegelhalder K, Johann A (2019). Insomnia as a predictor of mental disorders: a systematic review and meta-analysis. Sleep Med Rev.

[11] Reeve S, Sheaves B, Freeman D (2018). Sleep disorders in early psychosis: incidence, severity, and association with clinical symptoms. Schizophr Bull.

[12] Perry GS, Patil SP, Presley-Cantrell LR (2013). Raising awareness of sleep as a healthy behavior. Prev Chronic Dis.

[13] Lawrence D, Hancock KJ, Kisely S (2013). The gap in life expectancy from preventable physical illness in psychiatric patients in Western Australia: retrospective analysis of population based registers. BMJ (Clinical Research Ed).

[14] Cunningham R, Sarfati D, Stanley J, Peterson D, Collings S (2015). Cancer survival in the context of mental illness: a national cohort study. Gen Hosp Psychiatry.

[15] Firth J, Siddiqi N, Koyanagi A, Siskind D, Rosenbaum S, Galletly C (2019). The lancet psychiatry commission: a blueprint for protecting physical health in people with mental illness. Lancet Psychiatry.

[16] Franzen PL, Buysse DJ (2008). Sleep disturbances and depression: risk relationships for subsequent depression and therapeutic implications. Dialogues Clin Neurosci.

[17] Kallestad H, Hansen B, Langsrud K, Ruud T, Morken G, Stiles TC (2012). Impact of sleep disturbance on patients in treatment for mental disorders. BMC Psychiatry..

[18] Sheaves B, Freeman D, Isham L, McInerney J, Nickless A, Yu L-M (2018). Stabilising sleep for patients admitted at acute crisis to a psychiatric hospital (OWLS): an assessor-blind pilot randomised controlled trial. Psychol Med.

[19] Freeman D, Sheaves B, Goodwin GM, Yu L-M, Nickless A, Harrison PJ (2017). The effects of improving sleep on mental health (OASIS): a randomised controlled trial with mediation analysis. Lancet Psychiatry.

[20] Ben Simon E, Rossi A, Harvey AG, Walker MP. Overanxious and underslept. Nat Hum Behav. 2019. https://www.nature.com/articles/s41562-019-0754-8. 10.1038/s41562-019-0754-8 31685950

[21] Hirshkowitz M, Whiton K, Albert SM, Alessi C, Bruni O, DonCarlos L (2015). National Sleep Foundation's updated sleep duration recommendations: final report. Sleep Health..

[22] Ohayon M, Wickwire EM, Hirshkowitz M, Albert SM, Avidan A, Daly FJ (2017). National Sleep Foundation's sleep quality recommendations: first report. Sleep Health.

[23] Australasian Sleep Association. Consumers: Author; 2020 [Available from: https://www.sleep.org.au/Public/Resources/Consumers/Public/Resource-Centre/Consumers.aspx?hkey=43302712-12b2-4aa4-b575-c6301aa864e5.

[24] Metse AP, Bowman JA (2020). Prevalence of self-reported suboptimal sleep in Australia and receipt of sleep care: results from the 2017 National Social Survey. Sleep Health..

[25] National Prevention Council. National Prevention Strategy (2011). Washington, DC: U.S. Department of Health and Human Services, Office of the Surgeon General.

[26] Harris M, Llyod J (2012). The role of Australian primary health Care in the Prevention of chronic disease.

[27] The Royal Australian College of General Practitioners (2015). Smoking, nutrition, alcohol, physical activity (SNAP): A population health guide to behavioural risk factors in general practice (2nd edn.) Melbourne: Author.

[28] Lawrence D, Kisely S (2010). Inequalities in healthcare provision for people with severe mental illness. J Psychopharmacol.

[29] Solmi M, Firth J, Miola A, Fornaro M, Frison E, Fusar-Poli P (2020). Disparities in cancer screening in people with mental illness across the world versus the general population: prevalence and comparative meta-analysis including 4 717 839 people. Lancet Psychiatry.

[30] Department of Health Western Australia (2009). Sleep Disorders Model of Care. Perth: Health Networks Branch, Department of Health, Western Australia.

[31] Senthilvel E, Auckley D, Dasarathy J (2011). Evaluation of sleep disorders in the primary care setting: history taking compared to questionnaires. J Clin Sleep Med.

[32] O'Sullivan M, Rahim M, Hall C (2015). The prevalence and management of poor sleep quality in a secondary care mental health population. J Clin Sleep Med.

[33] Faulkner S, Bee P (2017). Experiences, perspectives and priorities of people with schizophrenia spectrum disorders regarding sleep disturbance and its treatment: a qualitative study. BMC Psychiatry..

[34] Waite F, Evans N, Myers E, Startup H, Lister R, Harvey AG (2016). The patient experience of sleep problems and their treatment in the context of current delusions and hallucinations. Psychol Psychother Theory Res Pract.

[35] Australian Bureau of Statistics (2015). General Social Survey: Summary Results, Australia, 2014 (CAT NO: 4159.0).

[36] Gordon S, Vandelanotte C, Rayward AT, Murawski B, Duncan MJ (2019). Sociodemographic and behavioral correlates of insufficient sleep in Australian adults. Sleep Health..

[37] Curtin R, Presser S, Singer E (2005). Changes in telephone survey nonresponse over the past quarter century. Public Opin Q.

[38] Stickley A, Leinsalu M, DeVylder JE, Inoue Y, Koyanagi A (2019). Sleep problems and depression among 237 023 community-dwelling adults in 46 low- and middle-income countries. Sci Rep.

[39] Hombali A, Seow E, Yuan Q, Chang SHS, Satghare P, Kumar S (2019). Prevalence and correlates of sleep disorder symptoms in psychiatric disorders. Psychiatry Res.

[40] Müller MJ, Olschinski C, Kundermann B, Cabanel N (2016). Subjective sleep quality and sleep duration of patients in a psychiatric hospital. Sleep Sci.

[41] Adams RJ, Appleton SL, Taylor AW, Gill TK, Lang C, McEvoy RD (2017). Sleep health of Australian adults in 2016: results of the 2016 sleep Health Foundation national survey. Sleep Health..

[42] Wittmann M, Dinich J, Merrow M, Roenneberg T (2006). Social jetlag: misalignment of biological and social time. Chronobiol Int.

[43] Appleton SL, Gill TK, Lang CJ, Taylor AW, McEvoy RD, Stocks NP (2018). Prevalence and comorbidity of sleep conditions in Australian adults: 2016 sleep Health Foundation national survey. Sleep Health..

[44] Talbot LS, Hairston IS, Eidelman P, Gruber J, Harvey AG (2009). The effect of mood on sleep onset latency and REM sleep in interepisode bipolar disorder. J Abnorm Psychol.

[45] Glozier N, O’Dea B, McGorry PD, Pantelis C, Amminger GP, Hermens DF (2014). Delayed sleep onset in depressed young people. BMC Psychiatry.

[46] American Psychiatric Association (2013). Diagnostic and statistical manual of mental disorders (5th ed.).

[47] Australian Institute of Health and Welfare (2018). Mental health services—in brief 2018. Cat. no. HSE 211.

[48] Alam A, Chengappa KNR (2011). Obstructive sleep apnoea and schizophrenia: a primer for psychiatrists. Acta Neuropsychiatrica.

[49] NIH State-of-the-Science Conference Statement on Manifestations and Management of Chronic Insomnia in Adults. NIH Consens Sci Statements. 2005;22(2):1–30. https://pubmed.ncbi.nlm.nih.gov/17308547/. 17308547

[50] Chung KF, Lee CT, Yeung WF, Chan MS, Chung EW, Lin WL. Sleep hygiene education as a treatment of insomnia: a systematic review and meta-analysis. Fam Pract. 2017. 10.1093/fampra/cmx122 29194467

[51] Ramakrishnan K, Scheid DC (2007). Treatment options for insomnia. Am Fam Physician.

[52] Rasu RS, Shenolikar RA, Nahata MC, Balkrishnan R (2005). Physician and patient factors associated with the prescribing of medications for sleep difficulties that are associated with high abuse potential or are expensive: an analysis of data from the National Ambulatory Medical Care Survey for 1996-2001. Clin Ther.

[53] Waters F, Chiu VW, Janca A, Atkinson A, Ree M (2015). Preferences for different insomnia treatment options in people with schizophrenia and related psychoses: a qualitative study. Front Psychol.

[54] Waters F, Bucks RS (2011). Neuropsychological effects of sleep loss: implication for neuropsychologists. J Int Neuropsychol Soc.

[55] The Royal Australian College of General Practitioners (RACGP) (2015). Smoking, nutrition, alcohol, physical activity (SNAP): A population health guide to behavioural risk factors in general practice. 2nd ed.

[56] Harvey AG, Tang NKY (2012). (Mis) perception of sleep in insomnia: a puzzle and a resolution. Psychol Bull.

[57] Kurina LM, McClintock MK, Chen JH, Waite LJ, Thisted RA, Lauderdale DS (2013). Sleep duration and all-cause mortality: a critical review of measurement and associations. Ann Epidemiol.

[58] Kronholm E, Laatikainen T, Peltonen M, Sippola R, Partonen T (2011). Self-reported sleep duration, all-cause mortality, cardiovascular mortality and morbidity in Finland. Sleep Med.

[59] Bei B, Milgrom J, Ericksen J, Trinder J (2010). Subjective perception of sleep, but not its objective quality, is associated with immediate postpartum mood disturbances in healthy women. Sleep..

[60] Moriarty DG, Zack MM, Kobau R (2003). The Centers for Disease Control and Prevention's healthy days measures - population tracking of perceived physical and mental health over time. Health Qual Life Outcomes.

[61] Johnson DA, Billings ME, Hale L (2018). Environmental determinants of insufficient sleep and sleep disorders: implications for population health. Curr Epidemiol Rep.

[62] Vleeshouwers J, Knardahl S, Christensen JO (2016). Effects of psychological and social work factors on self-reported sleep disturbance and difficulties initiating sleep. Sleep..

