# Correction to: Self-reported suboptimal sleep and receipt of sleep assessment and treatment among persons with and without a mental health condition in Australia: a cross sectional

**DOI:** 10.1186/s12889-021-10685-0

**Published:** 2021-04-01

**Authors:** Alexandra P. Metse, Caitlin Fehily, Tara Clinton-McHarg, Olivia Wynne, Sharon Lawn, John Wiggers, Jenny A. Bowman

**Affiliations:** 1 University of Newcastle, University Drive, Callaghan, NSW 2308 Australia; 2 University of the Sunshine Coast, 90 Sippy Downs Drive, Sippy Downs, QLD 4556 Australia; 3 Hunter Medical Research Institute, Lot 1 Kookaburra Circuit, New Lambton Heights, NSW 2305 Australia; 4 Murdoch University, 90 South Street, Murdoch, WA 6150 Australia; 5 Flinders University, Sturt Rd, Bedford Park, SA 5042 Australia; 6 Hunter New England Population Health, Longworth Avenue, Wallsend, NSW 2287 Australia

**Correction to: BMC Public Health (2021) 21:463**

**https://doi.org/10.1186/s12889-021-10504-6**

It was highlighted that in the original article [1] the addresses of affiliations 2 and 4 were erroneously interchanged. The original article has been updated.
