# Supplementary material for: Self-reported suboptimal sleep and receipt of sleep assessment and treatment among persons with and without a mental health condition in Australia: a cross sectional study
Source: BMC Public Health. 2021 Mar 6;21:463. doi: 10.1186/s12889-021-10504-6 (PMC7937198; doi:10.1186/s12889-021-10504-6)
Supplement: Supplementary file 1 — Additional file 1: Supplementary Table 1. NSF Criteria for Categorising Sleep Parameters as 'Appropriate', 'May be appropriate' and 'Suboptimal', According to Age. [file 12889_2021_10504_MOESM1_ESM.docx]

| **Supplementary Table 1. NSF Criteria for Categorising Sleep Parameters as 'Appropriate', 'May be appropriate' and 'Suboptimal', According to Age** | | | | |
| --- | --- | --- | --- | --- |
|  |  |  |  |  |
| **Sleep parameter** | **Age** | **Appropriate** | **May be appropriate** | **Suboptimal** |
| **Sleep duration (hours)** | 18-25 | 7-9 | 6, 10-11 | < 6, >11 |
|  | 26-64 | 7-9 | 6, 10 | < 6, >10 |
|  | 65+ | 7-8 | 5-6, 9 | <5, >9 |
|  |  |  |  |  |
| **Sleep onset latency (minutes)** | 18-25 | ≤30 | >30 ≤45 | >45 |
|  | 26-64 | ≤30 | >30 ≤45 | >45 |
|  | 65+ | ≤30 | >30 ≤60 | > 60 |
|  |  |  |  |  |
| **Awakenings (>5min)** | 18-25 | ≤1 | >1 ≤3 | >3 |
|  | 26-64 | ≤1 | >1 ≤3 | >3 |
|  | 65+ | ≤2 | >2 ≤3 | >3 |
|  |  |  |  |  |
| **Wake after sleep onset (minutes)** | 18-25 | ≤20 | >20 ≤40 | >40 |
|  | 26-64 | ≤20 | >20 ≤40 | >40 |
|  | 65+ | ≤30 | >30 | N/A |
|  |  |  |  |  |
| **Sleep efficiency (%)** | 18-25 | ≥85 | <85 ≥65 | <65 |
|  | 26-64 | ≥85 | <85 ≥75 | <75 |
|  | 65+ | ≥85 | <85 ≥75 | <75 |
|  |  |  |  |  |
| **Naps per day (n)** | 18-25 | 0 | 1-2 | >2 |
|  | 26-64 | N/A | 0-3 | >3 |
|  | 65+ | N/A | 0-3 | >3 |
|  |  |  |  |  |
| **Nap duration (minutes)** | 18-25 | N/A | 0-100 | >100 |
|  | 26-64 | N/A | 0-100 | >100 |
|  | 65+ | N/A | 0-100 | >100 |
|  |  |  |  |  |
| **Nap frequency (days)** | 18-25 | 0 | ≥1 | N/A |
|  | 26-64 | N/A | N/A | N/A |
|  | 65+ | N/A | N/A | N/A |
| N/A: not applicable, no 'suboptimal' criteria.  Table source: (24) | | | | |
